# Supplementary material for: Attachment and trauma-informed programme to support forcibly displaced parents of youth in Sweden: feasibility and preliminary outcomes of the eConnect Online programme
Source: BMJ Open. 2023 Aug 7;13(8):e072368. doi: 10.1136/bmjopen-2023-072368 (PMC10407377; doi:10.1136/bmjopen-2023-072368)
Supplement: Author's manuscript [file bmjopen-2023-072368.draft_revisions.pdf]

BMJ Open is committed to open peer review. As part of this commitment we make the peer review history of every article we publish publicly available.

When an article is published we post the peer reviewers' comments and the authors' responses online. We also post the versions of the paper that were used during peer review. These are the versions that the peer review comments apply to.

The versions of the paper that follow are the versions that were submitted during the peer review process. They are not the versions of record or the final published versions. They should not be cited or distributed as the published version of this manuscript.

BMJ Open is an open access journal and the full, final, typeset and author-corrected version of record of the manuscript is available on our site with no access controls, subscription charges or pay-per-view fees (http://bmjopen.bmj.com).

If you have any questions on BMJ Open's open peer review process please email info.bmjopen@bmj.com

BMJ Open

Delivering an Online Attachment and Trauma Focused Parenting Support Program for Forcibly Displaced Parents: Adaptations and Feasibility

Journal:	*BMJ Open*
Manuscript ID	bmjopen-2023-072368
Article Type:	Original research
Date Submitted by the Author:	31-Jan-2023

Complete List of Authors:	Kristen, Anna; Simon Fraser University,<br>Salari, Raziye; Uppsala Universitet Institutionen for folkhalso- och vardvetenskap, Child Health and Parenting, Department of Public Health and Caring Sciences<br>Moretti, Marlene; Simon Fraser University<br>Osman, Fatumo; Hogskolan Dalarna, School of Education, Health and Social Studies; Karolinska Institutet Department of Women's and Children's Health, Department of Women's and Children's health

Keywords:	Feasibility Studies, Health Services Accessibility, MENTAL HEALTH

SCHOLARONE™
Manuscripts

I, the Submitting Author has the right to grant and does grant on behalf of all authors of the Work (as defined in the below author licence), an exclusive licence and/or a non-exclusive licence for contributions from authors who are: i) UK Crown employees; ii) where BMJ has agreed a CC-BY licence shall apply, and/or iii) in accordance with the terms applicable for US Federal Government officers or employees acting as part of their official duties; on a worldwide, perpetual, irrevocable, royalty-free basis to BMJ Publishing Group Ltd ("BMJ") its licensees and where the relevant Journal is co-owned by BMJ to the co-owners of the Journal, to publish the Work in this journal and any other BMJ products and to exploit all rights, as set out in our licence.

The Submitting Author accepts and understands that any supply made under these terms is made by BMJ to the Submitting Author unless you are acting as an employee on behalf of your employer or a postgraduate student of an affiliated institution which is paying any applicable article publishing charge ("APC") for Open Access articles. Where the Submitting Author wishes to make the Work available on an Open Access basis (and intends to pay the relevant APC), the terms of reuse of such Open Access shall be governed by a Creative Commons licence – details of these licences and which Creative Commons licence will apply to this Work are set out in our licence referred to above.

Other than as permitted in any relevant BMJ Author's Self Archiving Policies, I confirm this Work has not been accepted for publication elsewhere, is not being considered for publication elsewhere and does not duplicate material already published. I confirm all authors consent to publication of this Work and authorise the granting of this licence.

For peer review only

Delivering an Online Attachment and Trauma Focused Parenting Support Program for Forcibly Displaced Parents: Adaptations and Feasibility

Anna Kristen,[1] Raziye Salari,[2] Marlene M. Moretti,[1] & Fatumo Osman [3]

[1] *Simon Fraser University, Burnaby, Canada;* [2] *Child Health and Parenting (CHAP), Department of Public Health and Caring Sciences, Uppsala University, Uppsala, Sweden;* [3] *Dalarna University*

Correspondence for this article should be addressed to Dr. Fatumo Osman: fos@du.se.

For peer review only

Abstract

Objectives: To assess the feasibility, acceptability, and the impact of an online parenting program for forcibly displaced parents of adolescents. **Design:** The study was a single-arm feasibility study using pre-intervention, post-intervention, and follow-up assessments. **Setting:** Participants were recruited from municipality-based activities for refugee parents in a small city in the south of Sweden. **Participants:** Participants were forcibly displaced parents (*n*=23) of youth (n=23; 8-17 years old) from Syria, Afghanistan, and Somalia participating in an online parenting program (*eConnect*). **Intervention:** *eConnect* is an attachment-based and trauma-informed parenting intervention and was delivered over the course of 10, weekly sessions. **Primary and secondary outcome measures:** Feasibility was assessed by program enrolment, attendance, retention, and acceptability of the online platform and cultural fit of the program. Primary outcome measures were reduction in mental health problems in youth. Secondary outcome measures were program impact on family functioning and parent-child attachment insecurity. **Results:** The *eConnect* program was highly feasible in terms of overall enrolment (100%), attendance (89.6%), and retention rates (100%). The online platform acceptable, with mixed feedback primarily related to the access and usage of technology. Cultural fit of the program was acceptable. Youth mental health problems ($\eta2$=0.29) and family functioning significantly improved ($\eta2$=0.18) over the course of the program. Unexpectedly, parent reports of youth attachment insecurity significantly worsened ($\eta2$=0.16). **Conclusions:** The findings suggest that the online delivery of *Connect* was a promising way to reduce barriers to service access and improve mental health problems and family functioning among forcibly displaced parents and their children during COVID-19. Future research is needed to explore the

For peer review only

acceptability and impact of this program post-COVID-19, and to develop culturally tailored and psychometrically sound measures parent and youth reports of attachment.

Strengths and limitations of this study

- Use of mixed methodologies including quantitative and qualitative data.
- High levels of participation and retention of culturally diverse parents in intervention study including 2-months follow-up.
- Unique multi-cultural sample of forcibly displaced parents resettled in Sweden.
- Small sample size and lack of control group.
- Attachment parent-report measure require validation in culturally diverse refugee samples.

For peer review only

Introduction

Globally, we are witnessing unprecedented rates of forced displacement due to violence, conflict, and human rights violations.[1] At the end of 2021, the forcibly displaced population exceeded 89 million, with over 40% being youth under the age of 18 years old.[1] In Sweden, there are currently over 250 thousand forcibly displaced persons, many of whom have fled from ongoing conflicts in Syria, Afghanistan, and Somalia.[2] Forcibly displaced families often experience significant pre-displacement trauma, including political conflict, war-related violence, and dangerous circumstances during migration.[3] Upon migration, families face significant acculturative stress including unfamiliar social norms and expectations, experiences of systemic racism and discrimination, and extreme deprivation of resources.[4 5] Familial acculturative stress is associated with poor family functioning,[6] disconnection within the parent-child relationship,[7] and heightened youth vulnerability for poor mental health outcomes.[8]

Forcibly displaced youth are at high risk of developing mental health disorders.[9] Adolescence is a period of heightened period of vulnerability for the onset of emotional and behaviour problems, and challenges related to assimilating or integration of cultural identity into their host culture is associated with poor mental health outcomes.[10] Indeed, a recent meta-analysis revealed that posttraumatic stress, depressive disorders, and anxiety disorders are highly prevalent among refugee youth who have resettled in European countries (53%, 33%, and 32% respectively).[11]

Evidence-based parenting interventions are known to address youth mental health problems and enhance caregiving practices.[12 13] More specifically, emerging evidence has demonstrated that evidence-based parenting interventions adapted to address the unique needs of forcibly displaced parents may be effective in improving family outcomes such as reducing

For peer review only - http://bmjopen.bmj.com/site/about/guidelines.xhtml

youth mental health problems, [14 15] and promoting family cohesion.[16 17] Yet, forcibly displaced parents often experience exceptionally poor access to healthcare services, due to limited access and awareness of service availability; perceived stigma around mental health problems; and fear of judgement, discrimination, or child removal by service providers.[18] Service access is further complicated for these families by the limited availability of culturally aligned services delivered in their native languages.[19]

The COVID-19 pandemic exacerbated challenges with healthcare access for families across the globe. Low-income populations, including forcibly displaced families, were disproportionately impacted by the challenges of COVID-19 given the pre-existing barriers they experienced in accessing healthcare services.[20] In several countries, public health restrictions precipitated a rapid and widespread shift to virtually delivered healthcare services. Yet, we lack knowledge of how to develop and implement culturally and contextually tailored parent support services for underserved populations; the barriers and facilitators of program uptake in these populations; and the impact of services in promoting parent, family, and youth mental health.

Beyond COVID-19, virtually delivered interventions have been shown to overcome other key barriers to service access (e.g., demanding work schedules).[21] Thus, the use of virtually delivered interventions may be a promising avenue to reduce service access disparity for underserved populations. A recent qualitative study exploring refugee experiences of virtually delivered mental health services highlighted that while access to technology posed a barrier to access, online service delivery eliminated other barriers to service access (e.g., transportation costs), and created new possibilities for culturally and contextually aligned service delivery in parents' natives languages.[22] Despite the potential for virtually delivered services to improve service access for forcibly displaced communities, there is a complete absence of research

For peer review only

examining the feasibility of virtually delivered parenting interventions for forcibly displaced families.

The Current Study

The *Connect* program is an evidence-based intervention designed for parents and alternative caregivers of youth ages 8-18 years old.[23] In previous studies, we adapted and implemented *Connect* for Somali-born parents in Sweden. The results demonstrated that the *Connect* program was effective for improving both parental and youth mental health problems, and for improving parental efficacy and satisfaction, and these treatment gains were maintained at a 3-year follow-up.[15 17] The current study aims to assess the feasibility, acceptability, and the impact of the online version of the *Connect* parenting program, *eConnect Online*[24] for forcibly displaced parents in Sweden.

Methods

Design and Study Population

The study is a part of a larger international research program evaluating the impact of an online version of a parenting program, *Connect*, on youth mental health.[24] The current study was a single-arm feasibility study using pre-intervention, post-intervention, and follow-up assessments. We recruited the parents from municipality-based activities serving refugee parents (e.g., languages classes) in a small city in the south of Sweden from May until August 2021. Parents were invited to an informational session where they received information about the program and the study. Both parents from families were encouraged to attend the program. All parents with youth aged 8-18 years old were eligible to participate. We emphasized that participation in the research study was voluntary and would not impact participation in the *Connect* program. Of the 30 parents who attended the informational session, all enrolled in the

For peer review only - http://bmjopen.bmj.com/site/about/guidelines.xhtml

eConnect Online program. Of these 23, (76.7%) consented to participate in the research study. All parents completed written informed consent for participation. Ethical approval was received from the Swedish Ethical Review Authority (Dnr. 2020-06737) and Simon Fraser University Office of Research Ethics (#2011s0284 and #20200401).

Patient and Public Involvement

Over the last 20 years, the *Connect* program has been recursively shaped in response to families' feedback. In this study, continuous discussions were carried out with the municipality and Humana organization for implementation of *eConnect*. Additionally, caregivers were invited to describe their experience and provide feedback at the end of the program.

Intervention

The *Connect* program is a manualized, attachment-based, and trauma-informed intervention for caregivers of youth aged 8-18 years old.[23] Through the use of experiential role plays and reflective exercises, the program is designed to promote caregiver reflective functioning and sensitivity to strengthen the caregiver-teen relationship. Extensive research has provided evidence for the effectiveness of *Connect* in reducing serious youth mental health problems.[12 25] The program is delivered by two trained facilitators over the course of 10, weekly 90-minutes sessions.

eConnect Online was adopted given in-person gathering restrictions due to the COVID-19 pandemic. The online version of *Connect* mirrors all program components (i.e., principles, role plays, reflective exercises, real-time flip charts) and real-time interactive group processes components of the in-person version of the program.[24] To support real-time interactions, *eConnect* utilizes a tech support person who supports parents' access to the online space and facilitates group exercises.

For peer review only

Prior to the first session, the group facilitators held a technology orientation session in which parents were introduced to and practiced using the videoconferencing platform. If needed, a limited numbers of computers were available that parents to borrow. In total, four *eConnect* facilitators, two females and two males, delivered the three groups included in this study. Three of them had previous experience in delivering the *Connect* program in-person. All four facilitators had lived experience as forcibly displaced immigrants from Somalia, Iraq, and Afghanistan.

In previous evaluations of the adaptation of the *Connect* program for Somali-speaking parents, the procedure included two initial group sessions focused on providing tailored societal information to parents such as information on the role of social services in Sweden.[15] In this study, *eConnect* was delivered in Somali, Dari, and Arabic languages. In the current study, societal information was provided to newcomer parents prior to the *eConnect* program. *eConnect* is flexible to cultural and contextual diversities. While the core attachment-based principles and components (e.g., role plays, reflection exercises) of *Connect* were retained, the group facilitators adjusted the role play content to align with the cultural context of each group.[26] For example, role play content was adjusted to capture conflict that commonly arises in displaced families related to acculturative tensions between child desire for cultural integration and autonomy and parental desire to uphold cultural values and practices, including respect for parental authority.[Error! Bookmark not defined.] The facilitators also used metaphors and proverbs to explain aspects of attachment principles such as empathy and conflict. These modifications provided opportunities for parents to reflect on, discuss, and practice different ways of integrating cultural beliefs and values with responsive and strength-focused parenting practices.

Data Collection Procedure

For peer review only

Parents were invited to complete self-report measures at pre-intervention (T1), at the end of the program (T2), and two-months following the completion of the program (T3). Most of the parents came to the municipality to complete the assessments where they were supported by group facilitators and interpreters. The lead researcher was present via Microsoft Teams to support completion of research materials. Parents were additionally invited to partake in focus group interviews at the end of the program, facilitated by the lead researcher via Microsoft Teams. All parents received honorarium for completing research materials equivalent to $10 USD at each time-point.

Feasibility and Acceptability Assessments

Feasibility of the intervention was assessed by program enrolment, attendance, and retention. For the current study, feasibility was determined by participant attendance of at least 7 program sessions. Acceptability of the virtual platform and cultural fit were assessed in the post-treatment group interviews. Parents additionally rated their familiarity before starting the program with videoconferencing platforms at post-intervention.

Outcome Measurements

Primary outcomes were reductions in mental health problems in youth. The secondary outcomes included improved family functioning and reductions in parent-child attachment insecurity.

Demographics

Parents also reported on sociodemographic information including their country of origin, age, highest level of education, and employment status.

Youth Mental Health Problems

For peer review only

Youth mental health problems were assessed using the Brief Child and Family Phone Interview (BCFPI).[27] The BCFPI is a 39-item standardized caregiver-report screening tool used to assess youth emotional and behavioural problems. We used the externalizing subscale (Attention-Deficit/Hyperactivity Disorder, Oppositional Defiant Disorder, and Conduct Disorder, six items each) and the internalizing subscale (Separation Anxiety Disorder, Generalized Anxiety Disorder, Major Depressive Disorder, six items each). We additionally calculated a total problem score (externalizing and internalizing scores combined). Parents rated the frequency of behaviours (pre-treatment baseline: over the past six months at T1; post-treatment: over the past two weeks at T2, and follow-up: over the past 2 months at T3). Items were rated on a 3-point Likert scale (1=*never*, 2=*sometimes*, 3=*often*). We additionally used the family functioning subscale that consists of six items rated on a 4-point Likert scale (1=*strongly agree*; 4=*strongly disagree*). The BCFPI has excellent psychometric properties.[28] In this study, the subscales showed good internal consistency at pre-intervention ($\alpha >= 0.81$).

Parent-Child Attachment Insecurity

Parent-child attachment insecurity was assessed using the caregiver-report version of a brief form of the Adolescent Attachment Anxiety & Avoidance Inventory (AAAAI).[23] The AAAAI consists of two subscales assessing attachment anxiety (seven items) and attachment avoidance (nine items). Items are scored on a 7-point Likert scale (1=*strongly disagree* to 7=*strongly agree*). We additionally calculated a total attachment insecurity score (anxiety and avoidance scores combined). Factor analytic research on the AAAAI has demonstrated strong psychometric properties.[23] In the current study, internal consistencies were acceptable (α ranging from 0.66 to .75).

Statistical Analyses

For peer review only - http://bmjopen.bmj.com/site/about/guidelines.xhtml

Statistical analyses were conducted using SPSS version 27.[29] Descriptive statistics were used for summarising pre-intervention demographic data, program attendance, and retention rates (n=23). To assess intervention effect on outcomes, one-way repeated measure analysis of variance (ANOVAs) was conducted to determine within-subject change over time from pre-intervention to post-intervention and 2-month follow-up. Of the 23 parents, one did not complete the follow-up assessment and was excluded from these analyses. We first conducted omnibus tests to determine whether there were significant mean differences overall between the timepoints for the study outcomes. The effect sizes of the overall differences were determined using an eta-squared calculation (η^2=.01-.06 small effect, η^2=.06-.14 medium effect, η^2>.14 large effect). When omnibus tests were significant (p<0.05), we tested for patterns of change (i.e., linear, and quadratic) over time.

Results

Participant Demographics

The sample included biological parents (n=23; 47.8% maternal figures; aged 28-64) of adolescents (n=23; 26.1% female; aged 8-17) who were forcibly displaced and living in Sweden. The parents reported their country of origins to be Afghanistan (n=10; 43.5%), Somalia (n=8; 34.8%), and Syria (n=5; 21.7%). Table 1 outlines additional demographic information.

Table 1. *Pre-intervention demographic characteristics of sample*

Variables	
Parents, n (%)	
Mothers	11(47.8)
Fathers	12 (52.2)
Participant age in years (mean±SD)	47.09±8.34
Years spent in host country (mean±SD)	7.63±7.58
Highest education level, n (%)	

For peer review only

No formal education	5(22.7)
Partial/completed primary school	9(40.9)
Partial/completed high school	6(27.2)
Apprenticeship/trades certificate	1 (4.5)
College/university completion	1(4.5)
Financial Well-Being, n (%)	
Not enough money to cover living expenses	2(9.1)
Barely enough money to cover living expenses	12(54.4)
Enough money to cover living expenses	8(36.4)
Civic status, n (%)	
Married	21(91.3)
Single	2(8.7)
Employment status: Unemployed, n (%)	18(90)
Multiple caregiver homes, n (%)	19(82.6)
No. of children in house (mean±SD)	4.70±2.03
Child sex: Female, n (%)	6(26.1)
Child age (mean±SD)	13.22±2.56
Child living in the parents' home, n (%)	23(100)
Child attending regular school, n (%)	23(100)

Feasibility: Enrolment, Attendance, and Retention

In total, 30 parents were invited to attend the *eConnect* program and all 30 parents enrolled in the program, demonstrating successful uptake. Out of the 30 parents attending, 23 parents (76.7%) consented to enroll in the research study. Three program groups were completed between September and December 2021: one for Somali-speaking parents, one for Dari-speaking parents, and one for Arabic-speaking parents. All parents attended between 7 to 10 sessions (M=8.87, SD=1.06). Retention rates (100%) across the three groups additionally demonstrate successful uptake.

Acceptability of the Online Platform

For peer review only

Online Delivery

We intended to deliver the groups online, with parents and facilitators joining from their respective locations on their respective computers. In the Arabic group, the program ran online as anticipated. The Somali group also ran online as anticipated; most joined from home except two parents who joined on their computers from the municipality due to internet instability at home and challenges using the videoconferencing platform. For the Dari group, adjustments to the delivery method had to be made after the first session. The online delivery was adjusted to a hybrid delivery due to parents' challenges with accessing stable internet. In this group, all parents attended in-person at the municipality office, and the two group facilitators joined online.

Familiarity with Technology and Accessibility of Online Delivery

At post-intervention, 17 parents rated their level of familiarity with videoconferencing platforms prior to beginning the group. Of these, 50% were somewhat familiar with videoconferencing, 11.1% had used videoconferencing platforms before but were not familiar with the technology, and 38.9% had never used videoconferencing platforms before.

In the post-intervention focus group interview, all parents (*n*=23) reported about their experiences with the online program delivery, including the advantages and disadvantages. Overall, the Arabic-speaking group (*n*=5) shared positive feedback, reporting on the many advantages of the online program delivery. For this group, the online delivery facilitated the attendance of both parents from each family and eliminated stressors related to being away from the home (e.g., childcare). The parents also enjoyed the comfort that came with participating from their home (e.g., having a cup of tea). The group reported that the online delivery of the roleplays was excellent, and the online delivery did not interfere with feeling "seen" by the group facilitators.

For peer review only

From the Somali-speaking group (*n*=8), some parents reported technology problems that sometimes made it challenging to attend the group. One parent reported feeling confused by the technology components. In addition, some parents reported that they would have preferred the interactions and connections that comes with being in-person. Despite technology challenges, the parents reported that they were still able to fully engage in the group discussions. One participant acknowledged that without the option for online delivery, attending the program would not have been possible given the circumstances of COVID-19.

The Dari-speaking group (*n*=10) reported a number of technology challenges. Some of the parents were unable to access a computer or stable internet. Those that were able to access the necessary technology reported difficulties using the videoconferencing platform. The parents reported having no previous experience in using online learning methods. The parents reported that being together in-person was central to their learning. For example, the parents noted that distractions at home (e.g., children) could get in the way of engaging in the program and retaining information. Additionally, they noted the advantages of being in-person in terms of connecting with one another. The parents agreed that the roleplays delivered online by facilitators were important to their learning, and the delivery of this was not impeded by the online platform.

Acceptability of Program Cultural Fit

Parents additionally provided feedback regarding the cultural fit of the program during the post-intervention focus group interviews. All three groups reported that it was essential that the group facilitators had lived experiences as immigrants and as parents. The parents explained that having group facilitators support them and truly understand their concerns as parents was contingent upon the group facilitators having experienced the pain and challenges themselves.

This lived experience was also essential for the group facilitators to accurately adapt the roleplays to the everyday challenges that the parents encounter at home with their teenagers, and all three groups reported that the role-plays did capture these challenges.

Intervention Outcomes

The within-subject changes from pre-intervention to post-intervention and 2-month follow-up are presented in Table 2.

Youth Mental Health Problems

For parent reports of total problems on the BCFPI, the repeated measures ANOVA revealed an overall significant effect with large effect sizes (η2=0.29). There were both significant linear and quadric components, indicating that total problems significantly decreased from pre-intervention (*M*=9.36, *SD*=2.11) to post-intervention (*M*=7.85, *SD*=1.61), and then remained almost the same from post-intervention to 2-month follow-up (*M*=7.88, *SD*=1.76).

A similar pattern of results was found for youth externalizing problems, with large effect sizes (η2=0.27). Parents reported fewer externalizing problems overtime (*M*=4.83, *SD*=1.36; *M*=3.73, *SD*=0.92; *M*=3.92, *SD*=0.10) over the three time points respectively), but the greatest reduction occurred from pre-intervention to post-intervention. For internalizing behaviors, the repeated measure ANOVA revealed no significant overall changes across the three timepoints.

Family Functioning

The repeated measures ANOVA revealed an overall significant effect on family functioning, with large effect sizes (η2=0.18). The linear component was significant while the quadric component was not, indicating that family functioning improved linearly over time from pre-intervention (*M*=1.64, *SD*=0.64) to post-intervention (*M*=1.34, *SD*=0.25) and from post-intervention to 2-month follow-up (*M*=1.27, *SD*=0.34).

For peer review only

Parent-Child Attachment Insecurity

The repeated measures ANOVA revealed an overall significant effect on parent-child attachment (η2=0.16). The linear component was significant while the quadric component was not. Unexpectedly, results showed attachment insecurity worsened linearly over time from pre-intervention (*M*=2.55, *SD*=0.79) to post-intervention (*M*=2.99, *SD*=1.00) to 2-month follow-up (*M*=3.18*, SD*=1.06). A similar pattern of results was found for parent-youth attachment anxiety subscale (η2 =0.16), with large effect sizes. Parents report steady increase in attachment anxiety over the course of the intervention from pre-intervention (*M*=3.07, *SD*=1.27) to post-intervention (*M*=3.58, *SD*=1.47), and to 2-month follow-up (*M*=3.97, *SD*=1.47). There were no significant overall changes in attachment avoidance across the three timepoints.

Table 2. *Within-group comparison of program parents at pre-intervention, post-intervention, and 2-month follow-up (n=22)*

				Overall		Linear		Quadratic	
Outcome Measure	Pre-intervention mean (SD)	Post-intervention mean (SD)	Follow-up mean (SD)	*F(1,21)*	η2	*F(1,21)*	η2	*F(1,21)*	η2
BCFPI									
Externalizing	4.83(1.36)	3.73(0.92)	3.92(0.10)	7.63*	0.27	9.88**	*.32*	5.71*	*.21*
Internalizing	4.52(1.06)	4.12(1.13)	3.95(0.94)	2.53	0.11	-	-		-
Total Difficulties	9.36(2.11)	7.85(1.61)	7.88(1.76)	8.60**	0.29	10.24**	.33	5.94*	.22
Family Functioning	1.64 (0.64)	1.34(0.25)	1.27(0.34)	4.53*	0.18	5.46*	.21	1.90	.08
AAAAIY									
Avoidance	2.02(0.84)	2.40(1.02)	2.39(1.02)	1.21	0.06	-	-	-	-
Anxiety	3.07(1.27)	3.58(1.47)	3.97(1.47)	3.91*	0.16	6.98*	.25	0.04	0
Insecurity	2.55(0.79)	2.99(1.00)	3.18(1.06)	4.12*	0.16	6.09*	.23	0.55	.03

Note. *p<0.05, **p<0.01, ***p<0.001. Lower scores on the BCFPI sub-scales indicate reductions in internalizing and externalizing problems, and improved family functioning. Lower scores on the AAAIY sub-scales indicate reductions in attachment insecurity (anxiety and avoidance).

For peer review only

Discussion

This single-arm study was conducted to test the feasibility, acceptability, and impact of an online attachment-based program for forcibly displaced parents of adolescents. We found that the *eConnec*t program was feasible with strong uptake reflected in enrollment (100%), attendance (89.6%), and retention of parents (100%) across the three groups. Parent feedback indicated *eConnect* enabled both parents from families to attend the sessions. More than 50% of the parents were fathers. This is a notable finding as fathers are typically underrepresented across parenting interventions,[30 31] despite the critical impact that father parenting practices have on positive youth mental health.[32] Prior research has also called for the inclusion of both parents in intervention work whenever possible to target multiple risk factors related to youth adverse outcomes (i.e., mother and father parenting practices).[33] The *eConnect* program also reduced barriers to service access and eliminated obstacles related to being away from home (e.g., childcare), which is of importance given the significant barriers to service access forcibly displaced families face.[18]

The online delivery of the program was acceptable to most of the parents. Although *eConnect* offered a means for forcibly displaced people to access parenting support during COVID-19, a lack of access to technology (i.e., computers and stable internet) as well as lack of familiarity with using videoconferencing platforms was a major challenge for some parents. In our study, the Dari-speaking parents were generally newer migrants, compared to the other groups, and thus they likely had less exposure to technology through the Swedish labor market. Indeed, the majority Dari-speaking parents reported that they had never used a videoconferencing platform before.

For peer review only

In terms of lack of access to technology, previous research conducted during COVID-19 has shown that lower household income is associated with lower levels of digital literacy and lower rates of access to virtual healthcare.[22 34] Our findings, and the high rates of poverty among forcibly displaced families, underscores the need for service provider support in facilitating access to technology for parents, and support in increasing parental familiarity with videoconferencing platforms for successful implementation. This seems particularly crucial for displaced parents that are newer to their host country.

Despite these technology challenges, the high rates of attendance and retention emphasize that parents were able to engage in the program, indicating that *eConnect* is a promising avenue for reducing service access disparity. We believe that the online delivery the *Connect* program can make it possible for municipalities or organizations to use facilitators who are geographically distant. Our findings additionally emphasize the importance of shared language and lived experience between the group facilitators and participant for parental engagement.[27] This finding might call attention to the need for culturally sensitive trainings for facilitators from non-migrant backgrounds for effective delivery.

We also completed a preliminary evaluation of the impact of *eConnect* Online on youth mental health problems and family functioning. Consistent with other research,[23 15 17] our findings indicated that the youths' total mental health problems, including externalizing problems, significantly declined from pre-intervention to post-intervention, and therapeutic gains were maintained at the two-month follow-up. While youths' internalizing problems did not significantly decline, the mean change scores suggest a reduction in internalizing problems. This finding could reflect the less observable nature of internalizing symptoms,[35] compared to externalizing symptoms. This finding was also present in our previous study,[15] in which we did

For peer review only - http://bmjopen.bmj.com/site/about/guidelines.xhtml

not detect a significant change in internalizing problems from pre-intervention and two-month follow-up but did observe a significant change in the long-term follow-up.[17] Consistent with prior research, family functioning significantly improved from pre-intervention to post-intervention, and post-intervention to two-month follow-up.

Unexpectedly, our findings showed parents reported significantly higher rather than lower levels of attachment insecurity over the course of the intervention. This is surprising in light of the significant improvements in youth mental health symptoms and family functioning (e.g., improved emotional closeness, openness in communication), and inconsistent with previous studies showing reductions in parent and youth reported levels of attachment insecurity following *Connect*.[12 36] It is important to note that the parent-report measure of attachment used in this study has not been previously validated within the cultural diverse and forcibly displaced families who participated in this research. Even though the measure was translated into the different languages of parents in this study, anecdotally it was observed that most parents struggled to understand the meaning of the items. It is possible that the meaning of these items differs for parents from these cultures due to differences in parenting goals and family values (e.g., emphasis on youth independence vs. family interdependence).[37] Qualitative research may be needed to support a better understanding of the language and concepts that capture parent-child attachment for forcibly displaced parents.

Strengths and Limitations

Our feasibility study has several limitations. First, while we were able to retain all parents in the program, our sample was small, and we had no control group. Second, even though all families who participated in the study shared a common history of forcible displacement, there may have been differences between their cultural backgrounds that we were unable to explore due to our

For peer review only

small sample. We consider this research to be a valuable first step, however, given the fact that forcibly displaced populations are greatly underrepresented in intervention research. Third, the attachment measure (AAAAI) used in this study was not validated for this population, and future work could investigate how forcibly displaced families conceptualize and understand attachment to inform the development and adaptation of self-report attachment measures. Future research should also include both parent and youth reports. Despite these limitations, this is the first study to our knowledge to evaluate the online delivery of a parenting intervention for forcibly displaced parents. The positive changes in youth mental health can also be seen as strength, given the high rates of mental health problems and high parental stress during COVID-19.[38]

Conclusion

The findings suggest that *eConnect* is a promising way to reduce barriers to service access and improve youth mental health problems and family functioning beyond COVID-19. This study underscores the important logistical considerations for the implementation of *eConnect* for forcibly displaced families, particularly the importance for service providers to consider how to support parents in accessing and feeling confident using the necessary technology. Service providers should also consider the expressed importance of the cultural and lived experiences of facilitators. Our findings emphasize the need for research to explore how forcibly displaced families conceptualize parent-child attachment in order to inform the development or adaptation of self-report measures for this population.

For peer review only

Patient consent for publication: Not applicable. All participants completed written informed consent to participate in the study.

Ethical Approval: Ethical approval was received from the Swedish Ethical Review Authority (Dnr. 2020-06737) and Simon Fraser University Office of Research Ethics (#2011s0284 and #20200401).

Contributorship statement: FO and MMM conceptualised the study. MMM is the developer of the *Connect* program. FO designed the study and led data collection. AK prepared the study data, and AK and RS performed the statistical analysis. All authors contributed to the interpretation of results. AK wrote the first draft of the manuscript. FO, RS, and MMM contributed to critical revision of the manuscript. All authors approved the final version before submission. The corresponding author attests that all listed authors meet authorship criteria and that no others meeting the criteria have been omitted. FO is the guarantor of the study.

Funding Statement: This work was supported by the Canadian Institutes of Health Research grant number #448851.

Competing Interest Statement: The authors report there are no conflicts of competing interests to declare.

Data sharing statement: The data that support the findings of this study are available on request from the corresponding author. The data are not publicly available due to privacy or ethical restrictions.

Acknowledgments: This work was completed with the support of a Canada Research Chair - Tier 1 in Youth Clinical Psychological Science. The authors would like to thank all the parents who participated in this study. We also would like to thank all the group facilitators who delivered the intervention and assisted us with the recruitment. Finally, we thank the Humana and Växjö municipality for the technical support.

For peer review only

References

1. Refugee statistics. UNHCR Canada. https://www.unhcr.ca/in-canada/refugee-statistics/. Published August 18, 2022.
2. Sweden refugee statistics 1960-2022. MacroTrends. https://www.macrotrends.net/countries/SWE/sweden/refugee-statistics.
3. Vossoughi, N., Jackson, Y., Gusler, S., & Stone, K. (2018). Mental Health Outcomes for Youth Living in Refugee Camps: A Review. *Trauma, Violence, & Abuse*, *19*(5), 528-542. https://doi.org/10.1177/1524838016673602
4. Osman, F., Klingberg-Allvin, M., Flacking, R., & Schön, U. K. (2016). Parenthood in transition - Somali-born parents' experiences of and needs for parenting support programmes. *BMC International Health and Human Rights*, *16*, 7. https://doi.org/10.1186/s12914-016-0082-2
5. Baghdasaryan, Z., Lampa, E., & Osman, F. (2021). 'Let us understand each other and work together in the child's best interest'–Exploring the narratives of newly arrived refugee parents in Sweden. *International Journal of Intercultural Relations*, *81*, 226-235. https://doi.org/10.1016/j.ijintrel.2021.02.004
6. Lorenzo-Blanco, E. I., Meca, A., Piña-Watson, B., Zamboanga, B. L., Szapocznik, J., Cano, M. Á., Cordova, D., Unger, J. B., Romero, A., Des Rosiers, S. E., Soto, D. W., Villamar, J. A., Pattarroyo, M., Lizzi, K. M., & Schwartz, S. J. (2019). Longitudinal trajectories of family functioning among recent immigrant adolescents and parents: Links with adolescent and parent cultural stress, emotional well-being, and behavioral health. *Child Development*, *90*(2), 506-523. https://doi.org/10.1111/cdev.12914
7. Betancourt, T. S., Abdi, S., Ito, B. S., Lilienthal, G. M., Agalab, N., Ellis, H. (2015) We left one war and came to another: Resource loss, acculturative stress, and caregiver–child

For peer review only

relationships in Somali refugee families. *Cultural Diversity and Ethnic Minority Psychology, 21*(1), 114-125. http://doi.org/10.1037/a0037538

8. d'Abreu, A., Castro-Olivo, S., & Ura, S. K. (2019). Understanding the role of acculturative stress on refugee youth mental health: A systematic review and ecological approach to assessment and intervention. *School Psychology International*, *40*(2), 107-127. https://doi.org/10.1177/0143034318822688
9. Blackmore, R., Boyle, J. A., Fazel, M., Ranasinha, S., Gray, K. M., Fitzgerald, G., Misso, M., & Gibson-Helm, M. (2020). The prevalence of mental illness in refugees and asylum seekers: A systematic review and meta-analysis. *PLoS Medicine*, *17*(9), e1003337. https://doi.org/10.1371/journal.pmed.1003337
10. Lincoln, A. K., Lazarevic, V., White, M. T., & Ellis, B. H. (2016). The impact of acculturation style and acculturative hassles on the mental health of somali adolescent refugees. *Journal of Immigrant and Minority Health*, *18*(4), 771-778. https://doi.org/10.1007/s10903-015-0232-y
11. Kien, C., Sommer, I., Faustmann, A., Gibson, L., Schneider, M., Krczal, E., Jank, R., Klerings, I., Szelag, M., Kerschner, B., Brattström, P., & Gartlehner, G. (2019). Prevalence of mental disorders in young refugees and asylum seekers in European Countries: A systematic review. *European Child & Adolescent Psychiatry*, *28*(10), 1295-1310. https://doi.org/10.1007/s00787-018-1215-z
12. Moretti, M. M., Obsuth, I., Craig, S. G., & Bartolo, T. (2015). An attachment-based intervention for parents of adolescents at risk: Mechanisms of change. *Attachment & Human Development*, *17*(2), 119-135. https://doi.org/10.1080/14616734.2015.1006383

13. Florean, I. S., Dobrean, A., Păsărelu, C. R., Georgescu, R. D., & Milea, I. (2020). The efficacy of internet-based parenting programs for children and adolescents with behavior problems: A meta-analysis of randomized clinical trials. *Clinical Child and Family Psychology Review*, *23*(4), 510-528. https://doi.org/10.1007/s10567-020-00326-0

14. Gillespie, S., Banegas, J., Maxwell, J., Chan, A. C. Y., Darawshy, N. A., Wasil, A. R., Marsalis, S., & Gewirtz, A. (2022). Parenting interventions for refugees and forcibly displaced families: A systematic review. *Clinical Child and Family Psychology Review*, *25*(2), 395-412. https://doi.org/10.1007/s10567-021-00375-z

15. Osman, F., Salari, R., Klingberg-Allvin, M., Schön, U. K., & Flacking, R. (2017). Effects of a culturally tailored parenting support programme in Somali-born parents' mental health and sense of competence in parenting: A randomized controlled trial. *BMJ Open*, *7*(12), e017600.

16. Minhas, R., & Benipal, P. (2018). Parenting interventions for refugee families: A systematic scoping review. *Paediatrics & Child Health*, *23*(suppl_1), e36-e36. https://doi.org/10.1093/pch/pxy054.094

17. Osman, F., Randell, E., Mohamed, A., & Sorbring, E. (2021). Dialectical processes in parent-child relationships among Somali families in Sweden. *Journal of Child and Family Studies*, *30*(7), 1752-1762. https://doi.org/10.1007/s10826-021-01956-w

18. Thomson, M. S., Chaze, F., George, U., & Guruge, S. (2015). Improving immigrant populations' access to mental health services in Canada: A review of barriers and recommendations. *Journal of Immigrant and Minority Health*, *17*(6), 1895-1905. https://doi.org/10.1007/s10903-015-0175-3

For peer review only - http://bmjopen.bmj.com/site/about/guidelines.xhtml

19. Hamari, L., Konttila, J., Merikukka, M., Tuomikoski, A. M., Kouvonen, P., & Kurki, M. (2022). Parent support programmes for families who are immigrants: A scoping review. *Journal of Immigrant and Minority Health*, *24*(2), 506-525. https://doi.org/10.1007/s10903-021-01181-z

20. Elisabeth, M., Maneesh, P. S., & Michael, S. (2020). Refugees in Sweden during the covid-19 pandemic-the need for a new perspective on health and integration. *Frontiers in Public Health*, *8*, 574334. https://doi.org/10.3389/fpubh.2020.574334

21. Siegel, A., Zuo, Y., Moghaddamcharkari, N., McIntyre, R. S., & Rosenblat, J. D. (2021). Barriers, benefits and interventions for improving the delivery of telemental health services during the coronavirus disease 2019 pandemic: A systematic review. *Current Opinion in Psychiatry*, *34*(4), 434-443. https://doi.org/10.1097/YCO.0000000000000714

22. Hynie, M., Jaimes, A., Oda, A., Rivest-Beauregard, M., Perez Gonzalez, L., Ives, N., Ahmad, F., Kuo, B. C. H., Arya, N., Bokore, N., & McKenzie, K. (2022). assessing virtual mental health access for refugees during the covid-19 pandemic using the levesque client-centered framework: What have we learned and how will we plan for the future? *International Journal of Environmental Research and Public Health*, *19*(9), 5001. https://doi.org/10.3390/ijerph19095001

23. Moretti, M. M., & Obsuth, I. (2009). Effectiveness of an attachment-focused manualized intervention for parents of teens at risk for aggressive behaviour: The Connect Program. *Journal of adolescence*, *32*(6), 1347-1357. https://doi.org/10.1016/j.adolescence.2009.07.013

For peer review only

24. Lin, B., & Moretti, M. M. (2022; in press) eConnect: Implementation and preliminary evaluation of a virtually delivered attachment-based parenting intervention during COVID-19. *Attachment & Human Development.*

25. Pasalich, D. S., Craig, S. G., Goulter, N., O'Donnell, K. A., Sierra Hernandez, C., & Moretti, M. M. (2022). Patterns and predictors of different youth responses to attachment-based parent intervention. *Journal of Clinical Child and Adolescent Psychology*, *51*(5), 796-809. https://doi.org/10.1080/15374416.2021.1923022

26. Osman, F., Schön, U. K., Klingberg-Allvin, M., Flacking, R., & Tistad, M. (2022). The implementation of a culturally tailored parenting support programme for Somali immigrant parents living in Sweden: A process evaluation. *PloS one*, *17*(9), e0274430. https://doi.org/10.1371/journal.pone.0274430

27. Cunningham, C. E., Pettingill, P., & Boyle, M. (2006). *The Brief Child and Family Phone Interview (BCFPI-3): A computerized intake and outcome assessment tool*. Interviewers Manual. © McMaster University.

28. Cook, S., Leschied, A. W., St Pierre, J., Stewart, S. L., den Dunnen, W., & Johnson, A. M. (2013). BCFPI Validation for a High-risk High-needs Sample of Children and Youth Admitted to Tertiary Care. *Journal of the Canadian Academy of Child and Adolescent Psychiatry, 22*(2), 147–152.

29. IBM Corp. (2020). *IBM SPSS Statistics for Windows, Version 27.0.* Armonk, NY: IBM Corp.

30. Chuang, S. S., & Costigan, C. L. (2018). *Parental roles and relationships in immigrant families* (pp. 1-8). New York: Springer.

31. Tully, L. A., Piotrowska, P. J., Collins, D. A. J., Mairet, K. S., Black, N., Kimonis, E. R., Hawes, D. J., Moul, C., Lenroot, R. K., Frick, P. J., Anderson, V., & Dadds, M. R. (2017). Optimising child outcomes from parenting interventions: Fathers' experiences, preferences and barriers to participation. *BMC Public Health*, *17*(1), 550. https://doi.org/10.1186/s12889-017-4426-1

32. Barker, B., Iles, J. E., & Ramchandani, P. G. (2017). Fathers, fathering and child psychopathology. *Current Opinion in Psychology*, *15*, 87-92. https://doi.org/10.1016/j.copsyc.2017.02.015

33. Cowan, C. P., & Cowan, P. A. (2019). Enhancing parenting effectiveness, fathers' involvement, couple relationship quality, and children's development: Breaking down silos in family policy making and service delivery. *Journal of Family Theory & Review*, *11*(1), 92-111. https://doi.org/10.1111/jftr.12301

34. Darrat, I., Tam, S., Boulis, M., & Williams, A. M. (2021). Socioeconomic disparities in patient use of telehealth during the coronavirus disease 2019 surge. *JAMA Otolaryngology-- Head & Neck Surgery*, *147*(3), 287–295. https://doi.org/10.1001/jamaoto.2020.5161

35. De Los Reyes A. (2013). Strategic objectives for improving understanding of informant discrepancies in developmental psychopathology research. *Development and Psychopathology*, *25*(3), 669-682. https://doi.org/10.1017/S0954579413000096

36. Barone, L., Carone, N., Costantino, A., Genschow, J., Merelli, S., Milone, A., Polidori, L., Ruglioni, L., & Moretti, M. M. (2021). Effect of a parenting intervention on decreasing adolescents' behavioral problems via reduction in attachment insecurity: A

For peer review only - http://bmjopen.bmj.com/site/about/guidelines.xhtml

longitudinal, multicenter, randomized controlled trial. *Journal of Adolescence*, *91*, 82-96. https://doi.org/10.1016/j.adolescence.2021.07.008

37. Kagitcibasi, C. (2002). A model of family change in cultural context. *Online Readings in Psychology and Culture, 6*(3). https://doi.org/10.9707/2307-0919.1059

38. Essler, S., Christner, N., & Paulus, M. (2021). Longitudinal relations between parental strain, parent-child relationship quality, and child well-being during the unfolding COVID-19 pandemic. *Child Psychiatry and Human Development*, *52*(6), 995–1011. https://doi.org/120.1007/s10578-021-01232-4

For peer review only

STROBE 2007 (v4) Statement—Checklist of items that should be included in reports of *cohort studies*

Section/Topic	Item #	Recommendation	Reported on page #
**Title and abstract**	1	(*a*) Indicate the study's design with a commonly used term in the title or the abstract	1
		(*b*) Provide in the abstract an informative and balanced summary of what was done and what was found	2-3
**Introduction**			
Background/rationale	2	Explain the scientific background and rationale for the investigation being reported	4-5
Objectives	3	State specific objectives, including any prespecified hypotheses	6
**Methods**			
Study design	4	Present key elements of study design early in the paper	6
Setting	5	Describe the setting, locations, and relevant dates, including periods of recruitment, exposure, follow-up, and data collection	6-9
Participants	6	(*a*) Give the eligibility criteria, and the sources and methods of selection of participants. Describe methods of follow-up	6-7
		(*b*) For matched studies, give matching criteria and number of exposed and unexposed	n/a
Variables	7	Clearly define all outcomes, exposures, predictors, potential confounders, and effect modifiers. Give diagnostic criteria, if applicable	9-10
Data sources/ measurement	8*	For each variable of interest, give sources of data and details of methods of assessment (measurement). Describe comparability of assessment methods if there is more than one group	9-10
Bias	9	Describe any efforts to address potential sources of bias	19-20
Study size	10	Explain how the study size was arrived at	n/a
Quantitative variables	11	Explain how quantitative variables were handled in the analyses. If applicable, describe which groupings were chosen and why	11
Statistical methods	12	(*a*) Describe all statistical methods, including those used to control for confounding	11
		(*b*) Describe any methods used to examine subgroups and interactions	n/a
		(*c*) Explain how missing data were addressed	n/a
		(*d*) If applicable, explain how loss to follow-up was addressed	11
		(*e*) Describe any sensitivity analyses	n/a

For peer review only

**Results**			
Participants	13*	(a) Report numbers of individuals at each stage of study—eg numbers potentially eligible, examined for eligibility, confirmed eligible, included in the study, completing follow-up, and analysed	6-7; 11
		(b) Give reasons for non-participation at each stage	6-7;11
		(c) Consider use of a flow diagram	n/a
Descriptive data	14*	(a) Give characteristics of study participants (eg demographic, clinical, social) and information on exposures and potential confounders	11
		(b) Indicate number of participants with missing data for each variable of interest	11
		(c) Summarise follow-up time (eg, average and total amount)	9
Outcome data	15*	Report numbers of outcome events or summary measures over time	11-16
Main results	16	(*a*) Give unadjusted estimates and, if applicable, confounder-adjusted estimates and their precision (eg, 95% confidence interval). Make clear which confounders were adjusted for and why they were included	n/a
		(*b*) Report category boundaries when continuous variables were categorized	n/a
		(*c*) If relevant, consider translating estimates of relative risk into absolute risk for a meaningful time period	n/a
Other analyses	17	Report other analyses done—eg analyses of subgroups and interactions, and sensitivity analyses	n/a
**Discussion**			
Key results	18	Summarise key results with reference to study objectives	17-19
**Limitations**			
Interpretation	20	Give a cautious overall interpretation of results considering objectives, limitations, multiplicity of analyses, results from similar studies, and other relevant evidence	17-20
Generalisability	21	Discuss the generalisability (external validity) of the study results	19-20
**Other information**			
Funding	22	Give the source of funding and the role of the funders for the present study and, if applicable, for the original study on which the present article is based	21

*Give information separately for cases and controls in case-control studies and, if applicable, for exposed and unexposed groups in cohort and cross-sectional studies.

For peer review only

Note: An Explanation and Elaboration article discusses each checklist item and gives methodological background and published examples of transparent reporting. The STROBE checklist is best used in conjunction with this article (freely available on the Web sites of PLoS Medicine at http://www.plosmedicine.org/, Annals of Internal Medicine at http://www.annals.org/, and Epidemiology at http://www.epidem.com/). Information on the STROBE Initiative is available at www.strobe-statement.org.

For peer review only

BMJ Open

An Attachment and Trauma Informed Program to Support Forcibly Displaced Parents of Youth in Sweden: Feasibility and Preliminary Outcomes of the eConnect Online Program.

Journal:	*BMJ Open*
Manuscript ID	bmjopen-2023-072368.R1
Article Type:	Original research
Date Submitted by the Author:	18-Jun-2023
Complete List of Authors:	Kristen, Anna; Simon Fraser University,<br>Salari, Raziye; Uppsala Universitet Institutionen for folkhalso- och vardvetenskap, Child Health and Parenting, Department of Public Health and Caring Sciences<br>Moretti, Marlene; Simon Fraser University<br>Osman, Fatumo; Hogskolan Dalarna, School of Education, Health and Social Studies; Karolinska Institutet Department of Women's and Children's Health, Department of Women's and Children's health
**Primary Subject Heading**:	Mental health
Secondary Subject Heading:	Health services research
Keywords:	Feasibility Studies, Health Services Accessibility, MENTAL HEALTH, Adolescent

SCHOLARONE™
Manuscripts

I, the Submitting Author has the right to grant and does grant on behalf of all authors of the Work (as defined in the below author licence), an exclusive licence and/or a non-exclusive licence for contributions from authors who are: i) UK Crown employees; ii) where BMJ has agreed a CC-BY licence shall apply, and/or iii) in accordance with the terms applicable for US Federal Government officers or employees acting as part of their official duties; on a worldwide, perpetual, irrevocable, royalty-free basis to BMJ Publishing Group Ltd ("BMJ") its licensees and where the relevant Journal is co-owned by BMJ to the co-owners of the Journal, to publish the Work in this journal and any other BMJ products and to exploit all rights, as set out in our licence.

The Submitting Author accepts and understands that any supply made under these terms is made by BMJ to the Submitting Author unless you are acting as an employee on behalf of your employer or a postgraduate student of an affiliated institution which is paying any applicable article publishing charge ("APC") for Open Access articles. Where the Submitting Author wishes to make the Work available on an Open Access basis (and intends to pay the relevant APC), the terms of reuse of such Open Access shall be governed by a Creative Commons licence – details of these licences and which Creative Commons licence will apply to this Work are set out in our licence referred to above.

Other than as permitted in any relevant BMJ Author's Self Archiving Policies, I confirm this Work has not been accepted for publication elsewhere, is not being considered for publication elsewhere and does not duplicate material already published. I confirm all authors consent to publication of this Work and authorise the granting of this licence.

For peer review only

An Attachment and Trauma Informed Program to Support Forcibly Displaced Parents of Youth in Sweden: Feasibility and Preliminary Outcomes of the *eConnect Online* Program

Anna Kristen,[1] Raziye Salari,[2] Marlene M. Moretti,[1] & Fatumo Osman [3]

[1] *Simon Fraser University, Burnaby, Canada;* [2] *Child Health and Parenting (CHAP), Department of Public Health and Caring Sciences, Uppsala University, Uppsala, Sweden;* [3] *Dalarna University*

Correspondence for this article should be addressed to Dr. Fatumo Osman: fos@du.se.

For peer review only

Abstract

Objectives: To assess the feasibility, acceptability, and the impact of an online parenting program for forcibly displaced parents of adolescents. **Design:** The study was a single-arm feasibility study using pre-intervention, post-intervention, and follow-up assessments. **Setting:** Participants were recruited from municipality-based activities for refugee parents in a small city in the south of Sweden. **Participants:** Participants were forcibly displaced parents (*n*=23; 47.8% maternal figures) of youth (*n*=23; 8-17 years old; 26.1% female) from Syria, Afghanistan, and Somalia participating in an online parenting program (*eConnect*). **Intervention:** *eConnect* is an attachment-based and trauma-informed parenting intervention and was delivered over the course of 10, weekly sessions. **Primary and secondary outcome measures:** Feasibility was assessed by program enrolment, attendance, completion, and acceptability of the online platform and cultural fit of the program. Primary outcome measures were program impact on youth mental health problems. Secondary outcome measures were program impact on family functioning and parent-child attachment insecurity. **Results:** The *eConnect* program was highly feasible in terms of overall enrolment (100%), attendance (89.6%), and retention rates (100%). The online platform was acceptable, with mixed feedback primarily related to the access and usage of technology. Cultural fit of the program was acceptable. Youth mental health problems ($\eta 2=0.29$) and family functioning significantly improved ($\eta 2=0.18$) over the course of the program. Unexpectedly, parent reports of youth attachment insecurity significantly worsened ($\eta 2=0.16$). **Conclusions:** The findings suggest that the online delivery of *Connect* was a promising way to reduce barriers to service access and improve mental health problems and family functioning among forcibly displaced parents and their children during COVID-19. Future research is needed

For peer review only

to explore the acceptability and impact of this program post-COVID-19, and to develop culturally tailored and psychometrically sound measures parent and youth reports of attachment.

Strengths and limitations of this study

- Use of mixed methodologies including quantitative and qualitative data.
- High levels of participation and program completion of culturally diverse parents in intervention study including 2-months follow-up.
- Unique multi-cultural sample of forcibly displaced parents resettled in Sweden.
- Small sample size and lack of control group.
- Attachment parent-report measure require validation in culturally diverse refugee samples.

For peer review only

Introduction

Globally, we are witnessing unprecedented rates of forced displacement due to violence, conflict, and human rights violations.[1] At the end of 2021, the forcibly displaced population exceeded 89 million, with over 40% being youth under the age of 18 years old.[1] In Sweden, there are currently over 250 thousand forcibly displaced persons, many of whom have fled from ongoing conflicts in Syria, Afghanistan, and Somalia.[2] Forcibly displaced families often experience significant pre-displacement trauma, including political conflict, war-related violence, and dangerous circumstances during migration.[3] Upon migration, families face significant acculturative stress including unfamiliar social norms and expectations, experiences of systemic racism and discrimination, and extreme deprivation of resources.[4 5] Familial acculturative stress is associated with poor family functioning,[6] disconnection within the parent-child relationship,[7] and heightened youth vulnerability for poor mental health outcomes.[8]

Forcibly displaced youth are at high risk of developing mental health disorders.[9] Adolescence is a period of heightened vulnerability for the onset of emotional and behaviour problems, and challenges related to assimilating or integration of cultural identity into their host culture is associated with poor mental health outcomes.[10] Indeed, a recent meta-analysis revealed that posttraumatic stress, depressive disorders, and anxiety disorders are highly prevalent among refugee youth who have resettled in European countries (53%, 33%, and 32% respectively).[11]

Evidence-based parenting interventions are known to address youth mental health problems and enhance caregiving practices.[12 13] More specifically, emerging evidence has demonstrated that evidence-based parenting interventions adapted to address the unique needs of forcibly displaced parents may be effective in improving family outcomes such as reducing youth mental health problems, [14 15] and promoting family cohesion.[16 17] Yet, forcibly displaced

For peer review only

parents often experience exceptionally poor access to healthcare services, due to limited awareness of service availability; perceived stigma around mental health problems; and fear of judgement, discrimination, or child removal by service providers.[18] Service access is further complicated for these families by the limited availability of culturally aligned services delivered in their native languages.[19]

The COVID-19 pandemic exacerbated challenges with healthcare access for families across the globe. Low-income populations, including forcibly displaced families, were disproportionately impacted by the challenges of COVID-19 given the pre-existing barriers they experienced in accessing healthcare services.[20] In several countries, public health restrictions precipitated a rapid and widespread shift to virtually delivered healthcare services. Yet, we lack knowledge of how to develop and implement culturally and contextually tailored parent support services for underserved populations; the barriers and facilitators of program uptake in these populations; and the impact of services in promoting parent, family, and youth mental health.

Beyond COVID-19, virtually delivered interventions have been shown to overcome other key barriers to service access (e.g., demanding work schedules).[21] Thus, the use of virtually delivered interventions may be a promising avenue to reduce service access disparity for underserved populations. A recent qualitative study exploring refugee experiences of virtually delivered mental health services highlighted that while access to technology posed a barrier to service use, online service delivery eliminated other barriers to service access (e.g., transportation costs), and created new possibilities for culturally and contextually aligned service delivery in parents' native languages.[22] Despite the potential for virtually delivered services to improve service access for forcibly displaced communities, there is a complete absence of

For peer review only

research examining the feasibility of virtually delivered parenting interventions for forcibly displaced families.

The Current Study

The *Connect* program is an attachment-based intervention designed for parents and alternative caregivers of youth ages 8-18 years old.[23] In previous studies, we adapted and implemented *Connect* for Somali-born parents in Sweden. The results demonstrated that the *Connect* program was effective for improving both parental and youth mental health problems, and for improving parental efficacy and satisfaction, and these treatment gains were maintained at a 3-year follow-up.[15 17] The current study aims to assess the feasibility, acceptability, and the impact of the online version of the *Connect* parenting program, *eConnect Online*[24] for forcibly displaced parents in Sweden.

Methods

Design and Study Population

The current study is a part of a larger international research program evaluating the impact of an online version of a parenting program, *Connect*, on youth mental health during the COVID-19 pandemic.[24] The project included the preliminary evaluation of *eConnect* in a Canadian sample, and studies assessing the feasibility of the *eConnect* program in culturally diverse contexts (e.g., Mexico, South Africa). The current study was a single-arm feasibility study using pre-intervention, post-intervention, and follow-up assessments. We recruited the parents from municipality-based activities serving refugee parents (e.g., languages classes) in a small city in the south of Sweden from May until August 2021. Parents were invited to an informational session where they received information about the program and the study. Both parents from families were encouraged to attend the program. All parents with youth aged 8-18

For peer review only - http://bmjopen.bmj.com/site/about/guidelines.xhtml

years old were eligible to participate. We emphasized that participation in the research study was voluntary and would not impact participation in the *eConnect* program. All parents completed written informed consent for participation. Ethical approval was received from the Swedish Ethical Review Authority (Dnr. 2020-06737) and Simon Fraser University Office of Research Ethics (#2011s0284 and #20200401).

Patient and Public Involvement

Over the last 20 years, the *Connect* program has been recursively shaped in response to families' feedback. In this study, continuous discussions were carried out with the municipality and Humana organization for implementation of the *eConnect* program. Additionally, parents were invited to describe their experience and provide feedback at the end of the program.

Intervention

The Connect program is a manualized, attachment-based, and trauma-informed intervention for caregivers of youth aged 8-18 years old.[23] Through the use of experiential role plays and reflective exercises, the program is designed to promote caregiver reflective functioning and sensitivity to strengthen the caregiver-teen relationship. Extensive research has provided evidence for the effectiveness of *Connect* in reducing serious youth mental health problems.[12 25] The program is delivered by two trained facilitators over the course of 10, weekly 90-minutes sessions.

eConnect Online was adopted given in-person gathering restrictions due to the COVID-19 pandemic. The online version of *Connect* mirrors all program components (i.e., principles, role plays, reflective exercises, real-time flip charts) and real-time interactive group process components of the in-person version of the program.[24] To support real-time interactions, *eConnect* utilizes a tech support person who supports parents' access to the online space and

For peer review only

facilitates group exercises. The results from the preliminary evaluation of the *eConnect* program in Canada were highly comparable to research evaluating the in-person method of delivery.[24]

Prior to the first session, the group facilitators held a technology orientation session in which parents were introduced to, and practiced using, the videoconferencing platform. If needed, a limited numbers of computers were available for parents to borrow. In total, four *eConnect* facilitators (two females and two males) delivered the three groups included in this study. Three of the facilitators had previous experience in delivering the *Connect* program in-person. All four facilitators had lived experience as forcibly displaced immigrants from Somalia, Iraq, and Afghanistan.

In previous evaluations of the adaptation of the *Connect* program for Somali-speaking parents in Sweden, the procedure included two initial group sessions focused on providing tailored societal information to parents such as information on the role of social services in Sweden.[15] In the current study, societal information was provided to newcomer parents prior to the *eConnect* program. In this study, *eConnect* was delivered in Somali, Dari, and Arabic languages. *eConnect* is flexible to cultural and contextual diversities. While the core attachment-based principles and components (e.g., role plays, reflection exercises) of *Connect* were retained, the group facilitators adjusted the role play content to align with the cultural context of each group.[26] For example, role play content was adjusted to capture conflict that commonly arises in displaced families related to acculturative tensions between child desire for cultural integration and autonomy and parental desire to uphold cultural values and practices, including respect for parental authority.**Error! Bookmark not defined.** The facilitators also used metaphors and proverbs to explain aspects of attachment principles such as empathy and conflict. These modifications

For peer review only

provided opportunities for parents to reflect on, discuss, and practice different ways of integrating cultural beliefs and values with responsive and strength-focused parenting practices.

Data Collection Procedure

Parents were invited to complete self-report measures at pre-intervention (T1), at the end of the program (T2), and two-months following the completion of the program (T3). Most of the parents came to the municipality to complete the assessments where they were supported by group facilitators and interpreters. The lead researcher was also present via Microsoft Teams to support completion of research materials. Some of the parents completed research assessments at home and were encouraged to contact the lead researcher should any support be required. Parents were additionally invited to partake in focus group interviews (in their respective native languages) at the end of the program, facilitated by the lead researcher via Microsoft Teams. The lead researcher has extensive experience in facilitating focus group interviews, and is fluent in Somali, Arabic, Swedish, and English. A professional interpreter joined the Dari focus groups. To mitigate the potential for loss of meaning or accuracy in parents' descriptions during the interpretation process, the audio tape was also reviewed for accuracy by a professional transcriber fluent in Dari. All parents received honorarium for completing research materials equivalent to $10 USD at each time-point.

Feasibility and Acceptability Assessments

Feasibility of the intervention was assessed by program enrolment, attendance, and completion. For the current study, feasibility was determined by participant attendance of at least 7 program sessions. Acceptability of the virtual platform and cultural fit were assessed in the post-intervention group interviews. Parents additionally rated their familiarity before starting the program with videoconferencing platforms at post-intervention.

For peer review only

Outcome Measurements

Primary outcomes were reductions in mental health problems in youth. The secondary outcomes included improved family functioning and reductions in parent-child attachment insecurity.

Demographics

Parents also reported on sociodemographic information including their country of origin, age, highest level of education, and employment status.

Youth Mental Health Problems

Youth mental health problems were assessed using the Brief Child and Family Phone Interview (BCFPI).[27] The BCFPI is a 39-item standardized caregiver-report screening tool used to assess youth emotional and behavioural problems. We used the externalizing subscale (Attention-Deficit/Hyperactivity Disorder, Oppositional Defiant Disorder, and Conduct Disorder, six items each) and the internalizing subscale (Separation Anxiety Disorder, Generalized Anxiety Disorder, Major Depressive Disorder, six items each). We additionally calculated a total problem score (externalizing and internalizing scores combined). Parents rated the frequency of behaviours (pre-intervention baseline: over the past six months at T1; post-intervention: over the past two weeks at T2, and follow-up: over the past 2 months at T3). Items were rated on a 3-point Likert scale (1=*never*, 2=*sometimes*, 3=*often*). We additionally used the family functioning subscale that consists of six items rated on a 4-point Likert scale (1=*strongly agree*; 4=*strongly disagree*). The BCFPI has excellent psychometric properties.[28] In this study, the subscales showed good internal consistency at pre-intervention ($\alpha >= 0.81$).

Parent-Child Attachment Insecurity

For peer review only - http://bmjopen.bmj.com/site/about/guidelines.xhtml

Parent-child attachment insecurity was assessed using the caregiver-report version of a brief form of the Adolescent Attachment Anxiety & Avoidance Inventory (AAAAI).[23] The AAAAI consists of two subscales assessing attachment anxiety (seven items) and attachment avoidance (nine items). Items are scored on a 7-point Likert scale (1=*strongly disagree* to 7=*strongly agree*). We additionally calculated a total attachment insecurity score (anxiety and avoidance scores combined). Factor analytic research on the AAAAI has demonstrated strong psychometric properties.[23] In the current study, internal consistencies were acceptable (α ranging from 0.66 to .75).

Statistical Analyses

Statistical analyses were conducted using SPSS version 27.[29] Descriptive statistics were used for summarising program enrolment, attendance, and completion rates (n=30), and pre-intervention demographic data (n=23). To assess intervention effect on outcomes, one-way repeated measure analysis of variance (ANOVAs) was conducted to determine within-subject change over time from pre-intervention to post-intervention and 2-month follow-up. Of the 23 parents that consented to complete the study measures, one did not complete the follow-up assessment and was excluded from these analyses. We first conducted omnibus tests to determine whether there were significant mean differences overall between the timepoints for the study outcomes. The effect sizes of the overall differences were determined using an eta-squared calculation (η^2=.01-.06 small effect, η^2=.06-.14 medium effect, η^2>.14 large effect). We calculated the confidence intervals (CI) for effect sizes using the method suggested by Wuensch (2019).[30] When omnibus tests were significant (p<0.05), we tested for patterns of change (i.e., linear, and quadratic) over time.

Results

For peer review only

Participant Demographics

Of the 30 parents who attended the informational session, all parents enrolled in the *eConnect Online* program, including two couples. Three program groups were completed between September and December 2021: one for Somali-speaking parents, one for Dari-speaking parents, and one for Arabic-speaking parents. We collected session attendance and program completion rates for all 30 parents. However, to avoid dependency in the data, only one parent from each couple was eligible to complete the study outcome measures. Of the 28 parents eligible to participate in the research, 23 (82.1%) consented and were retained to complete study outcome measures.

The retained sample were biological parents (*n*=23; 47.8% maternal figures; aged 28-64) of adolescents (*n*=23; 26.1% female; aged 8-17) who were forcibly displaced and living in Sweden. The parents reported their country of origins to be Afghanistan (*n*=10; 43.5%), Somalia (*n*=8; 34.8%), and Syria (*n*=5; 21.7%). Table 1 outlines additional demographic information.

Table 1. *Pre-intervention demographic characteristics of sample*

Variables	
Parents, n (%)	
Mothers	11(47.8)
Fathers	12 (52.2)
Participant age in years (mean±SD)	47.09±8.34
Years spent in host country (mean±SD)	7.63±7.58
Highest education level, n (%)	
No formal education	5(22.7)
Partial/completed primary school	9(40.9)
Partial/completed high school	6(27.2)
Apprenticeship/trades certificate	1 (4.5)
College/university completion	1(4.5)

For peer review only

Financial Well-Being, n (%)	
Not enough money to cover living expenses	2(9.1)
Barely enough money to cover living expenses	12(54.4)
Enough money to cover living expenses	8(36.4)
Civic status, n (%)	
Married	21(91.3)
Single	2(8.7)
Employment status: Unemployed, n (%)	18(90)
Multiple caregiver homes, n (%)	19(82.6)
No. of children in house (mean±SD)	4.70±2.03
Child sex: Female, n (%)	6(26.1)
Child age (mean±SD)	13.22±2.56
Child living in the parents' home, n (%)	23(100)
Child attending regular school, n (%)	23(100)

Feasibility: Program Uptake and Completion

Across all three groups – for Somali-speaking, Dari-speaking, and Arabic-speaking parents – all parents who were invited to enrol in the program (*n*=30) attended between 7 to 10 sessions (*M*=8.87, *SD*=1.06) and completed the program (100%), showing excellent program uptake across all three groups.

Acceptability of the Online Platform

Online Delivery

We intended to deliver the groups online, with parents and facilitators joining from their respective locations on their respective computers. In the Arabic group, the program ran online as anticipated. The Somali group also ran online as anticipated; most joined from home except two parents who joined on their computers from the municipality due to internet instability at home and challenges using the videoconferencing platform. For the Dari group, adjustments to

For peer review only

the delivery method had to be made after the first session. The online delivery was adjusted to a hybrid delivery due to parents' challenges with accessing stable internet and necessary technology. In this group, all parents attended in-person at the municipality office, and the two group facilitators joined online.

Familiarity with Technology and Accessibility of Online Delivery

At post-intervention, 17 parents (74%) rated their level of familiarity with videoconferencing platforms prior to beginning the group. Of these, 50% were somewhat familiar with videoconferencing, 11.1% had used videoconferencing platforms before but were not familiar with the technology, and 38.9% had never used videoconferencing platforms before.

In the post-intervention focus group interview, all parents (*n*=23) reported about their experiences with the online program delivery, including the advantages and disadvantages. Overall, the Arabic-speaking group (*n*=5) shared positive feedback, reporting on the many advantages of the online program delivery. For this group, the online delivery facilitated the attendance of both parents from each family and eliminated stressors related to being away from the home (e.g., childcare). The parents also enjoyed the comfort that came with participating from their home (e.g., having a cup of tea). The group reported that the online delivery of the roleplays was excellent, and the online delivery did not interfere with feeling "seen" by the group facilitators.

From the Somali-speaking group (*n*=8), some parents reported technology problems that sometimes made it challenging to attend the group. One parent reported feeling confused by the technology components. In addition, some parents reported that they would have preferred the interactions and connections that comes with being in-person. Despite technology challenges, the parents reported that they were still able to fully engage in the group discussions. One participant

For peer review only

acknowledged that without the option for online delivery, attending the program would not have been possible given the circumstances of COVID-19.

The Dari-speaking group (*n*=10) reported a number of technology challenges. Some of the parents were unable to access a computer or stable internet. Those that were able to access the necessary technology reported difficulties using the videoconferencing platform. The parents reported having no previous experience in using online learning methods. The parents reported that being together in-person was central to their learning. For example, the parents noted that distractions at home (e.g., children) could get in the way of engaging in the program and retaining information. Additionally, they noted the advantages of being in-person in terms of connecting with one another. The parents agreed that the roleplays delivered online by facilitators were important to their learning, and the delivery of this was not impeded by the online platform.

Acceptability of Program Cultural Fit

Parents additionally provided feedback regarding the cultural fit of the program during the post-intervention focus group interviews. All three groups reported that it was essential that the group facilitators had lived experiences as immigrants and as parents. The parents explained that having group facilitators support them and truly understand their concerns as parents was contingent upon the group facilitators having experienced the pain and challenges themselves. This lived experience was also essential for the group facilitators to accurately adapt the roleplays to the everyday challenges that the parents encounter at home with their teenagers, and all three groups reported that the role-plays did capture these challenges.

Intervention Outcomes

For peer review only

The within-subject changes from pre-intervention to post-intervention and 2-month follow-up are presented in Table 2.

Youth Mental Health Problems

For parent reports of total problems on the BCFPI, the repeated measures ANOVA revealed an overall significant effect with large effect sizes (η2=0.29; 95% CI [0.09 to 0.43]). There were both significant linear and quadric components, indicating that total problems significantly decreased from pre-intervention (*M*=9.36, *SD*=2.11) to post-intervention (*M*=7.85, *SD*=1.61), and then remained almost the same from post-intervention to 2-month follow-up (*M*=7.88, *SD*=1.76).

A similar pattern of results was found for youth externalizing problems, with large effect sizes (η2=0.27; 95% CI [0.07 to 0.41]). Parents reported fewer externalizing problems overtime (*M*=4.83, *SD*=1.36; *M*=3.73, *SD*=0.92; *M*=3.92, *SD*=0.10, over the three timepoints respectively), but the greatest reduction occurred from pre-intervention to post-intervention. For internalizing behaviors, the repeated measure ANOVA revealed no significant overall changes across the three timepoints.

Family Functioning

The repeated measures ANOVA revealed an overall significant effect on family functioning, with large effect sizes (η2=0.18; 95% CI [0.02 to 0.32]). The linear component was significant while the quadric component was not, indicating that family functioning improved linearly over time from pre-intervention (*M*=1.64, *SD*=0.64) to post-intervention (*M*=1.34, *SD*=0.25) and from post-intervention to 2-month follow-up (*M*=1.27, *SD*=0.34).

Parent-Child Attachment Insecurity

For peer review only

The repeated measures ANOVA revealed an overall significant effect on parent-child attachment (η2=0.16; 95% CI [0.01 to 0.30]). The linear component was significant while the quadric component was not. Counter to predictions and prior research, results showed attachment insecurity worsened linearly over time from pre-intervention (*M*=2.55, *SD*=0.79) to post-intervention (*M*=2.99, *SD*=1.00) to 2-month follow-up (*M*=3.18, *SD*=1.06). A similar pattern of results was found for parent-youth attachment anxiety subscale (η2 =0.16; 95% CI [0.01 to 0.30]), with large effect sizes. Parents report steady increase in attachment anxiety over the course of the intervention from pre-intervention (*M*=3.07, *SD*=1.27) to post-intervention (*M*=3.58, *SD*=1.47), and to 2-month follow-up (*M*=3.97, *SD*=1.57). There were no significant overall changes in attachment avoidance across the three timepoints.

Table 2. *Within-group comparison of program parents at pre-intervention, post-intervention, and 2-month follow-up (n=22)*

					Overall			Linear			Quadratic	
Outcome Measure	Pre-intervention mean (SD)	Post-intervention mean (SD)	Follow-up mean (SD)		*F(2,42)*	η2		*F(1,21)*	η2		*F(1,21)*	η2
BCFPI												
Externalizing	4.83(1.36)	3.73(0.92)	3.92(0.10)		7.63*	0.27		9.88**	*0.32*		5.71*	*0.21*
Internalizing	4.52(1.06)	4.12(1.13)	3.95(0.94)		2.53	0.11		-	-		-	-
Total Difficulties	9.36(2.11)	7.85(1.61)	7.88(1.76)		8.60**	0.29		10.24**	0.33		5.94*	0.22
Family Functioning	1.64 (0.64)	1.34(0.25)	1.27(0.34)		4.53*	0.18		5.46*	0.21		1.90	0.08
AAAAIY												
Avoidance	2.02(0.84)	2.40(1.02)	2.39(1.02)		1.21	0.06		-	-		-	-
Anxiety	3.07(1.27)	3.58(1.47)	3.97(1.57)		3.91*	0.16		6.98*	0.25		0.04	0.00
Insecurity	2.55(0.79)	2.99(1.00)	3.18(1.06)		4.12*	0.16		6.09*	0.23		0.55	0.03

Note. *p<0.05, **p<0.01. Lower scores on the BCFPI sub-scales indicate reductions in internalizing and externalizing problems, and improved family functioning. Lower scores on the AAAIY sub-scales indicate reductions in attachment insecurity (anxiety and avoidance).

Discussion

This single-arm study was conducted to test the feasibility, acceptability, and impact of an online attachment-based program for forcibly displaced parents of adolescents. We found that the *eConnec*t program was feasible with strong uptake reflected in enrollment (100%), excellent attendance (89.6% of sessions completed), and exceptional program completion for all parents (100%) across the three groups. Parent feedback indicated *eConnect* enabled both parents from families to attend the sessions. More than 50% of the parents were fathers. This is a notable finding as fathers are typically underrepresented across parenting interventions,[31 32] despite the critical impact that father parenting practices have on positive youth mental health.[33] Prior research has also called for the inclusion of both parents in intervention work whenever possible to target multiple risk factors related to youth adverse outcomes (i.e., mother and father parenting practices).[34] The *eConnect* program also reduced barriers to service access and eliminated obstacles related to being away from home (e.g., childcare), which is of importance given the significant barriers to service access that forcibly displaced families face.[18]

The online delivery of the program was acceptable to most of the parents. Although *eConnect* offered a means for forcibly displaced people to access parenting support during COVID-19, a lack of access to technology (i.e., computers and stable internet) as well as lack of familiarity with using videoconferencing platforms was a major challenge for some parents, primarily the Dari-speaking parents. In our study, the Dari-speaking parents were generally newer migrants, compared to the other groups, and thus they likely had less exposure to technology through the Swedish labor market. Indeed, the majority Dari-speaking parents reported that they had never used a videoconferencing platform before.

For peer review only - http://bmjopen.bmj.com/site/about/guidelines.xhtml

In terms of lack of access to technology, previous research conducted during COVID-19 has shown that lower household income is associated with lower levels of digital literacy and lower rates of access to virtual healthcare.[22 35] Our findings, and the high rates of poverty among forcibly displaced families, underscore the need for service provider support in facilitating access to technology for parents, and support in increasing parental familiarity with videoconferencing platforms for successful implementation. This seems particularly crucial for displaced parents that are newer to their host country.

Despite these technology challenges, the high rates of program enrolment, attendance, and completion emphasize that parents were able to engage in the program, indicating that *eConnect* is a promising avenue for reducing service access disparity. Our findings additionally emphasize the importance of shared language and lived experience between the group facilitators and participants for parental engagement.[27] This finding might call attention to the need for culturally sensitive trainings for facilitators from non-migrant backgrounds for effective delivery. The current findings reveal the pressing need and eagerness among forcibly displaced parents to address complex caregiving challenges. Importantly, the online delivery the *Connect* program makes it possible for municipalities or organizations to use facilitators who are geographically distant, increasing program accessibility and cost-efficiency.

We also completed a preliminary evaluation of the impact of *eConnect* Online on youth mental health problems and family functioning. Consistent with recent research evaluating *eConnect* in Canada and other culturally diverse contexts, [23 36] our findings indicated that the youths' total mental health problems, including externalizing problems, significantly declined from pre-intervention to post-intervention, and therapeutic gains were maintained at the two-month follow-up. While youths' internalizing problems did not significantly decline, the mean

For peer review only

change scores suggest a reduction in internalizing problems. This finding could reflect our small sample size or the less observable nature of internalizing symptoms,[37] compared to externalizing symptoms. This finding was also present in our previous study,[15] in which we did not detect a significant change in internalizing problems from pre-intervention and two-month follow-up but did observe a significant change in the long-term follow-up (3 years).[17] Consistent with prior research, family functioning significantly improved from pre-intervention to post-intervention, and post-intervention to two-month follow-up.

Unexpectedly, our findings showed parents reported significantly higher rather than lower levels of attachment insecurity over the course of the intervention. This is surprising in light of the significant improvements in youth mental health symptoms and family functioning (e.g., emotional closeness, openness in communication), and inconsistent with previous studies showing reductions in parent and youth reported levels of attachment insecurity following participation in the *Connect*[12 38] and *eConnect* program. [23 36] It is important to note that the parent-report measure of attachment used in this study has not been previously validated within the culturally diverse and forcibly displaced families who participated in this research. Even though the measure was translated into the different languages of parents in this study, anecdotally it was observed that most parents struggled to understand the meaning of the items. It is possible that the meaning of these items differs for parents from these cultures due to differences in parenting goals and family values (e.g., emphasis on youth independence vs. family interdependence).[39] Alternatively, it is possible that forcibly displaced parents focus foremost on their child's tangible needs, risks, and safety given the magnitude of their displacement experiences. Youth themselves may also mask feelings of distress as not to further burden their parents. Qualitative research may be needed to support a better understanding of the

For peer review only

language and concepts that capture parent-child attachment for forcibly displaced parents, and the complex processes that may unfold in parent-child communication due to trauma.

Strengths and Limitations

Our feasibility study has several limitations. First, while all the parents completed the program, our sample was small, and we had no control group. Second, even though all families who participated in the study shared a common history of forcible displacement, there may have been differences between their cultural backgrounds that we were unable to explore due to our small sample. Third, even though the majority of the eligible parents (82%) consented to completing study outcome measures, we were not able to collect demographic information from the parents who did not consent. However, all parents recruited to participate in the program met the same inclusion criteria including parenting status, age of child, history of forcible displacement, and all parents were either Somali, Dari or Arabic speaking, so it is unlikely that those who participated or declined to do so differed in significant ways. Thus, despite our small sample size, we consider this research to be a valuable first step, given that forcibly displaced populations are greatly underrepresented in intervention research. Finally, the measure of youth mental health problems (BCFPI) and the attachment measure (AAAAI) used in this study were not validated for this population. However, the internal consistencies in this study showed acceptable levels. There is a significant need for future research to deeply examine how forcibly displaced families conceptualize and understand attachment to inform the development and adaptation of self-report attachment measures, and to investigate processes that underlie the impact of displacement trauma on parents, youth, and attachment. Future research should aim to include the voices of both parent and youth reports in both qualitative and quantitative methods of data collection. Despite these limitations, this is the first study to our knowledge to evaluate

For peer review only

the online delivery of a parenting intervention for forcibly displaced parents. The positive changes in youth mental health can also be seen as strength, given the high rates of mental health problems and high parental stress prior to and during COVID-19.[40]

Conclusion

The findings suggest that *eConnect* is a promising way to reduce barriers to service access and improve youth mental health problems and family functioning beyond COVID-19. This study underscores the important logistical considerations for the implementation of *eConnect* for forcibly displaced families, particularly the importance for service providers to consider how to support parents in accessing and feeling confident using the necessary technology. Service providers should also consider the expressed importance of the cultural and lived experiences of facilitators. Our findings emphasize the need for research to explore how forcibly displaced families conceptualize parent-child attachment in order to inform the development or adaptation of self-report measures for this population.

For peer review only

Patient consent for publication: Not applicable. All participants completed written informed consent to participate in the study.

Ethical Approval: Ethical approval was received from the Swedish Ethical Review Authority (Dnr. 2020-06737) and Simon Fraser University Office of Research Ethics (#2011s0284 and #20200401).

Contributorship statement: FO and MMM conceptualised the study. MMM is the developer of the *Connect* program. FO designed the study and led data collection. AK prepared the study data, and AK and RS performed the statistical analysis. All authors contributed to the interpretation of results. AK wrote the first draft of the manuscript. FO, RS, and MMM contributed to critical revision of the manuscript. All authors approved the final version before submission. The corresponding author attests that all listed authors meet authorship criteria and that no other authors meeting the criteria have been omitted. FO is the guarantor of the study.

Funding Statement: This work was supported by the Canadian Institutes of Health Research grant number #448851.

Competing Interest Statement: The authors report there are no conflicts of competing interests to declare.

Data sharing statement: The data that support the findings of this study are available on request from the corresponding author. The data are not publicly available due to privacy or ethical restrictions.

Acknowledgments: This work was completed with the support of a Canada Research Chair - Tier 1 in Youth Clinical Psychological Science. The authors would like to thank all the parents who participated in this study. We also would like to thank all the group facilitators who delivered the intervention and assisted us with the recruitment. Finally, we thank the Humana and Växjö municipality for the technical support.

For peer review only

References

1. Refugee statistics. UNHCR Canada. https://www.unhcr.ca/in-canada/refugee-statistics/. Published August 18, 2022.
2. Sweden refugee statistics 1960-2022. MacroTrends. https://www.macrotrends.net/countries/SWE/sweden/refugee-statistics.
3. Vossoughi, N., Jackson, Y., Gusler, S., & Stone, K. (2018). Mental health outcomes for youth living in refugee camps: A review. *Trauma, Violence, & Abuse*, *19*(5), 528-542. https://doi.org/10.1177/1524838016673602
4. Osman, F., Klingberg-Allvin, M., Flacking, R., & Schön, U. K. (2016). Parenthood in transition - Somali-born parents' experiences of and needs for parenting support programmes. *BMC International Health and Human Rights*, *16*, 7. https://doi.org/10.1186/s12914-016-0082-2
5. Baghdasaryan, Z., Lampa, E., & Osman, F. (2021). 'Let us understand each other and work together in the child's best interest'–Exploring the narratives of newly arrived refugee parents in Sweden. *International Journal of Intercultural Relations*, *81*, 226-235. https://doi.org/10.1016/j.ijintrel.2021.02.004
6. Lorenzo-Blanco, E. I., Meca, A., Piña-Watson, B., Zamboanga, B. L., Szapocznik, J., Cano, M. Á., Cordova, D., Unger, J. B., Romero, A., Des Rosiers, S. E., Soto, D. W., Villamar, J. A., Pattarroyo, M., Lizzi, K. M., & Schwartz, S. J. (2019). Longitudinal trajectories of family functioning among recent immigrant adolescents and parents: Links with adolescent and parent cultural stress, emotional well-being, and behavioral health. *Child Development*, *90*(2), 506-523. https://doi.org/10.1111/cdev.12914
7. Betancourt, T. S., Abdi, S., Ito, B. S., Lilienthal, G. M., Agalab, N., Ellis, H. (2015) We left one war and came to another: Resource loss, acculturative stress, and caregiver–child

For peer review only - http://bmjopen.bmj.com/site/about/guidelines.xhtml

relationships in Somali refugee families. *Cultural Diversity and Ethnic Minority Psychology, 21*(1), 114-125. http://doi.org/10.1037/a0037538

8. d'Abreu, A., Castro-Olivo, S., & Ura, S. K. (2019). Understanding the role of acculturative stress on refugee youth mental health: A systematic review and ecological approach to assessment and intervention. *School Psychology International*, *40*(2), 107-127. https://doi.org/10.1177/0143034318822688
9. Blackmore, R., Boyle, J. A., Fazel, M., Ranasinha, S., Gray, K. M., Fitzgerald, G., Misso, M., & Gibson-Helm, M. (2020). The prevalence of mental illness in refugees and asylum seekers: A systematic review and meta-analysis. *PLoS Medicine*, *17*(9), e1003337. https://doi.org/10.1371/journal.pmed.1003337
10. Lincoln, A. K., Lazarevic, V., White, M. T., & Ellis, B. H. (2016). The impact of acculturation style and acculturative hassles on the mental health of somali adolescent refugees. *Journal of Immigrant and Minority Health*, *18*(4), 771-778. https://doi.org/10.1007/s10903-015-0232-y
11. Kien, C., Sommer, I., Faustmann, A., Gibson, L., Schneider, M., Krczal, E., Jank, R., Klerings, I., Szelag, M., Kerschner, B., Brattström, P., & Gartlehner, G. (2019). Prevalence of mental disorders in young refugees and asylum seekers in European Countries: A systematic review. *European Child & Adolescent Psychiatry*, *28*(10), 1295-1310. https://doi.org/10.1007/s00787-018-1215-z
12. Moretti, M. M., Obsuth, I., Craig, S. G., & Bartolo, T. (2015). An attachment-based intervention for parents of adolescents at risk: Mechanisms of change. *Attachment & Human Development*, *17*(2), 119-135. https://doi.org/10.1080/14616734.2015.1006383

For peer review only

13. Florean, I. S., Dobrean, A., Păsărelu, C. R., Georgescu, R. D., & Milea, I. (2020). The efficacy of internet-based parenting programs for children and adolescents with behavior problems: A meta-analysis of randomized clinical trials. *Clinical Child and Family Psychology Review*, *23*(4), 510-528. https://doi.org/10.1007/s10567-020-00326-0

14. Gillespie, S., Banegas, J., Maxwell, J., Chan, A. C. Y., Darawshy, N. A., Wasil, A. R., Marsalis, S., & Gewirtz, A. (2022). Parenting interventions for refugees and forcibly displaced families: A systematic review. *Clinical Child and Family Psychology Review*, *25*(2), 395-412. https://doi.org/10.1007/s10567-021-00375-z

15. Osman, F., Salari, R., Klingberg-Allvin, M., Schön, U. K., & Flacking, R. (2017). Effects of a culturally tailored parenting support programme in Somali-born parents' mental health and sense of competence in parenting: A randomized controlled trial. *BMJ Open*, *7*(12), e017600.

16. Minhas, R., & Benipal, P. (2018). Parenting interventions for refugee families: A systematic scoping review. *Paediatrics & Child Health*, *23*(suppl_1), e36-e36. https://doi.org/10.1093/pch/pxy054.094

17. Osman, F., Randell, E., Mohamed, A., & Sorbring, E. (2021). Dialectical processes in parent-child relationships among Somali families in Sweden. *Journal of Child and Family Studies*, *30*(7), 1752-1762. https://doi.org/10.1007/s10826-021-01956-w

18. Thomson, M. S., Chaze, F., George, U., & Guruge, S. (2015). Improving immigrant populations' access to mental health services in Canada: A review of barriers and recommendations. *Journal of Immigrant and Minority Health*, *17*(6), 1895-1905. https://doi.org/10.1007/s10903-015-0175-3

For peer review only

19. Hamari, L., Konttila, J., Merikukka, M., Tuomikoski, A. M., Kouvonen, P., & Kurki, M. (2022). Parent support programmes for families who are immigrants: A scoping review. *Journal of Immigrant and Minority Health*, *24*(2), 506-525. https://doi.org/10.1007/s10903-021-01181-z

20. Elisabeth, M., Maneesh, P. S., & Michael, S. (2020). Refugees in Sweden during the covid-19 pandemic-the need for a new perspective on health and integration. *Frontiers in Public Health*, *8*, 574334. https://doi.org/10.3389/fpubh.2020.574334

21. Siegel, A., Zuo, Y., Moghaddamcharkari, N., McIntyre, R. S., & Rosenblat, J. D. (2021). Barriers, benefits and interventions for improving the delivery of telemental health services during the coronavirus disease 2019 pandemic: A systematic review. *Current Opinion in Psychiatry*, *34*(4), 434-443. https://doi.org/10.1097/YCO.0000000000000714

22. Hynie, M., Jaimes, A., Oda, A., Rivest-Beauregard, M., Perez Gonzalez, L., Ives, N., Ahmad, F., Kuo, B. C. H., Arya, N., Bokore, N., & McKenzie, K. (2022). assessing virtual mental health access for refugees during the covid-19 pandemic using the levesque client-centered framework: What have we learned and how will we plan for the future? *International Journal of Environmental Research and Public Health*, *19*(9), 5001. https://doi.org/10.3390/ijerph19095001

23. Moretti, M. M., & Obsuth, I. (2009). Effectiveness of an attachment-focused manualized intervention for parents of teens at risk for aggressive behaviour: The Connect Program. *Journal of adolescence*, *32*(6), 1347-1357. https://doi.org/10.1016/j.adolescence.2009.07.013

24. Lin, B., & Moretti, M. M. (2023) eConnect: Implementation and preliminary evaluation of a virtually delivered attachment-based parenting intervention during COVID-19.

For peer review only

Attachment & Human Development. 25(2), 272–288. https://doi.org/10.1080/14616734.2023.2179574

25. Pasalich, D. S., Craig, S. G., Goulter, N., O'Donnell, K. A., Sierra Hernandez, C., & Moretti, M. M. (2022). Patterns and predictors of different youth responses to attachment-based parent intervention. *Journal of Clinical Child and Adolescent Psychology*, *51*(5), 796-809. https://doi.org/10.1080/15374416.2021.1923022
26. Osman, F., Schön, U. K., Klingberg-Allvin, M., Flacking, R., & Tistad, M. (2022). The implementation of a culturally tailored parenting support programme for Somali immigrant parents living in Sweden: A process evaluation. *PloS one*, *17*(9), e0274430. https://doi.org/10.1371/journal.pone.0274430
27. Cunningham, C. E., Pettingill, P., & Boyle, M. (2006). *The Brief Child and Family Phone Interview (BCFPI-3): A computerized intake and outcome assessment tool*. Interviewers Manual. © McMaster University.
28. Cook, S., Leschied, A. W., St Pierre, J., Stewart, S. L., den Dunnen, W., & Johnson, A. M. (2013). BCFPI validation for a high-risk high-needs sample of children and youth admitted to tertiary care. *Journal of the Canadian Academy of Child and Adolescent Psychiatry, 22*(2), 147–152.
29. IBM Corp. (2020). *IBM SPSS Statistics for Windows, Version 27.0.* Armonk, NY: IBM Corp.
30. Wuensch, K. L. (2019) Putting Confidence Intervals on *R2*, h*2,* or *R ,* Retrieved from https://core.ecu.edu/wuenschk/StatHelp/CI-R2.htm on the 14th of June, 2023.
31. Chuang, S. S., & Costigan, C. L. (2018). *Parental roles and relationships in immigrant families* (pp. 1-8). New York: Springer.

32. Tully, L. A., Piotrowska, P. J., Collins, D. A. J., Mairet, K. S., Black, N., Kimonis, E. R., Hawes, D. J., Moul, C., Lenroot, R. K., Frick, P. J., Anderson, V., & Dadds, M. R. (2017). Optimising child outcomes from parenting interventions: Fathers' experiences, preferences and barriers to participation. *BMC Public Health*, *17*(1), 550. https://doi.org/10.1186/s12889-017-4426-1

33. Barker, B., Iles, J. E., & Ramchandani, P. G. (2017). Fathers, fathering and child psychopathology. *Current Opinion in Psychology*, *15*, 87-92. https://doi.org/10.1016/j.copsyc.2017.02.015

34. Cowan, C. P., & Cowan, P. A. (2019). Enhancing parenting effectiveness, fathers' involvement, couple relationship quality, and children's development: Breaking down silos in family policy making and service delivery. *Journal of Family Theory & Review*, *11*(1), 92-111. https://doi.org/10.1111/jftr.12301

35. Darrat, I., Tam, S., Boulis, M., & Williams, A. M. (2021). Socioeconomic disparities in patient use of telehealth during the coronavirus disease 2019 surge. *JAMA Otolaryngology-- Head & Neck Surgery*, *147*(3), 287–295. https://doi.org/10.1001/jamaoto.2020.5161

36. Benzi, I. M. A., Carone, N., Moretti, M., Ruglioni, L., Tracchegiani, J., & Barone, L. (2023). eCONNECT Parent Group: An online attachment-based intervention to reduce attachment insecurity, behavioral problems, and emotional dysregulation in adolescence. *International Journal of Environmental Research and Public Health*, *20*(4), 3532. https://doi.org/10.3390/ijerph20043532

37. De Los Reyes A. (2013). Strategic objectives for improving understanding of informant discrepancies in developmental psychopathology research. *Development and Psychopathology*, *25*(3), 669-682. https://doi.org/10.1017/S0954579413000096

For peer review only

38. Barone, L., Carone, N., Costantino, A., Genschow, J., Merelli, S., Milone, A., Polidori, L., Ruglioni, L., & Moretti, M. M. (2021). Effect of a parenting intervention on decreasing adolescents' behavioral problems via reduction in attachment insecurity: A longitudinal, multicenter, randomized controlled trial. *Journal of Adolescence*, *91*, 82-96. https://doi.org/10.1016/j.adolescence.2021.07.008

39. Kagitcibasi, C. (2002). A model of family change in cultural context. *Online Readings in Psychology and Culture, 6*(3). https://doi.org/10.9707/2307-0919.1059

40. Essler, S., Christner, N., & Paulus, M. (2021). Longitudinal relations between parental strain, parent-child relationship quality, and child well-being during the unfolding COVID-19 pandemic. *Child Psychiatry and Human Development*, *52*(6), 995–1011. https://doi.org/120.1007/s10578-021-01232-4

For peer review only

STROBE 2007 (v4) Statement—Checklist of items that should be included in reports of *cohort studies*

Section/Topic	Item #	Recommendation	Reported on page #
**Title and abstract**	1	(*a*) Indicate the study's design with a commonly used term in the title or the abstract	1
		(*b*) Provide in the abstract an informative and balanced summary of what was done and what was found	2-3
**Introduction**			
Background/rationale	2	Explain the scientific background and rationale for the investigation being reported	4-5
Objectives	3	State specific objectives, including any prespecified hypotheses	6
**Methods**			
Study design	4	Present key elements of study design early in the paper	6
Setting	5	Describe the setting, locations, and relevant dates, including periods of recruitment, exposure, follow-up, and data collection	6-9
Participants	6	(*a*) Give the eligibility criteria, and the sources and methods of selection of participants. Describe methods of follow-up	6-7
		(*b*) For matched studies, give matching criteria and number of exposed and unexposed	n/a
Variables	7	Clearly define all outcomes, exposures, predictors, potential confounders, and effect modifiers. Give diagnostic criteria, if applicable	9-10
Data sources/ measurement	8*	For each variable of interest, give sources of data and details of methods of assessment (measurement). Describe comparability of assessment methods if there is more than one group	9-10
Bias	9	Describe any efforts to address potential sources of bias	19-20
Study size	10	Explain how the study size was arrived at	n/a
Quantitative variables	11	Explain how quantitative variables were handled in the analyses. If applicable, describe which groupings were chosen and why	11
Statistical methods	12	(*a*) Describe all statistical methods, including those used to control for confounding	11
		(*b*) Describe any methods used to examine subgroups and interactions	n/a
		(*c*) Explain how missing data were addressed	n/a
		(*d*) If applicable, explain how loss to follow-up was addressed	11
		(*e*) Describe any sensitivity analyses	n/a

For peer review only

**Results**			
Participants	13*	(a) Report numbers of individuals at each stage of study—eg numbers potentially eligible, examined for eligibility, confirmed eligible, included in the study, completing follow-up, and analysed	6-7; 11
		(b) Give reasons for non-participation at each stage	6-7;11
		(c) Consider use of a flow diagram	n/a
Descriptive data	14*	(a) Give characteristics of study participants (eg demographic, clinical, social) and information on exposures and potential confounders	11
		(b) Indicate number of participants with missing data for each variable of interest	11
		(c) Summarise follow-up time (eg, average and total amount)	9
Outcome data	15*	Report numbers of outcome events or summary measures over time	11-16
Main results	16	(*a*) Give unadjusted estimates and, if applicable, confounder-adjusted estimates and their precision (eg, 95% confidence interval). Make clear which confounders were adjusted for and why they were included	n/a
		(*b*) Report category boundaries when continuous variables were categorized	n/a
		(*c*) If relevant, consider translating estimates of relative risk into absolute risk for a meaningful time period	n/a
Other analyses	17	Report other analyses done—eg analyses of subgroups and interactions, and sensitivity analyses	n/a
**Discussion**			
Key results	18	Summarise key results with reference to study objectives	17-19
**Limitations**			
Interpretation	20	Give a cautious overall interpretation of results considering objectives, limitations, multiplicity of analyses, results from similar studies, and other relevant evidence	17-20
Generalisability	21	Discuss the generalisability (external validity) of the study results	19-20
**Other information**			
Funding	22	Give the source of funding and the role of the funders for the present study and, if applicable, for the original study on which the present article is based	21

*Give information separately for cases and controls in case-control studies and, if applicable, for exposed and unexposed groups in cohort and cross-sectional studies.

For peer review only

Note: An Explanation and Elaboration article discusses each checklist item and gives methodological background and published examples of transparent reporting. The STROBE checklist is best used in conjunction with this article (freely available on the Web sites of PLoS Medicine at http://www.plosmedicine.org/, Annals of Internal Medicine at http://www.annals.org/, and Epidemiology at http://www.epidem.com/). Information on the STROBE Initiative is available at www.strobe-statement.org.

For peer review only
